# Epidemiology of children's swimming competence and water safety

**DOI:** 10.3389/fpubh.2022.961342

**Published:** 2022-07-22

**Authors:** Asier Santibañez-Gutierrez, Julen Fernández-Landa, Julio Calleja-González, Nikola Todorović, Marijana Ranisavljev, Valdemar Štajer, Bogdan Anđelić, Nataša Zenić, Antonino Bianco, Patrik Drid

**Affiliations:** ^1^Physical Education and Sports Department, Faculty of Education and Sport, University of the Basque Country (UPV/EHU), Vitoria-Gasteiz, Spain; ^2^Faculty of Sport and Physical Education, University of Novi Sad, Novi Sad, Serbia; ^3^Sport and Exercise Sciences Research Unit, University of Palermo, Palermo, Italy; ^4^Faculty of Kinesiology, University of Split, Split, Croatia

**Keywords:** school children, drowning, safety measures, swimming skills, aquatic events

## Abstract

**Introduction:**

The main purpose of this study was to investigate children's swimming competence in primary schools of districts in Vojvodina, Serbia.

**Methods:**

Included subjects were primary school students from first to eighth grade (*N* = 2,778; male = 1,454, female = 1,324; age = 10.73 ± 2.1 years). We used Swimming Competence Questionnaire to acquire and analyze their swimming experience, non-fatal aquatic events, and demographics. For the statistical analysis, logistic regression and hierarchical multiple regression were used to evaluate if the factors and SC and NFAE were associated. The analyses were carried out by using SPSS® software version 24.0 (SPSS, Inc., Chicago, Illinois, USA).

**Results:**

Families with more income and education generally have children with more swimming competence, experience, knowledge, and skills related to water safety. First step in analysis revealed that gender (β = 0.05, *p* < 0.01), education level (β = 0.06, *p* < 0.01) age (β = 0.171, *p* < 0.01), and family income (β = 0.04, *p* < 0.01) were significant swimming competence (SC) predictors (R2 = 0.04). Age (OR = 1.15, *p* < 0.01) was the only significant predictor in Step 1 predicting non-fatal aquatic events (NFAE). In Step 2, variables associated with SC were swimming location (ΔR2 = 0.06, *p* < 0.01), swimming experience (ΔR2 = 0.16, *p* < 0.01), swimming accessibility (ΔR2 = 0.05, *p* < 0.01), and learning experience (ΔR2 = 0.03, *p* < 0.01) (total R2 = 0.26 to 0.47, *p* < 0.01). Only a minority of participants reported that they could not swim further than 5 meters using general stroke (37.15%).

**Conclusion:**

National education trainers programs must be prioritized with the primary strategy of transferring knowledge to swimming and water safety. Families with lower income must be included without exceptions. This is perhaps a key factor in preventing NFAE, increasing SC, and increasing water safety.

## Introduction

Regular engagement in physical activity provides numerous acute and chronic beneficial effects to adolescents (1). Swimming is characterized as a lifelong and low-impact activity, regarded as one of the most popular among children (2). Both aquatic activities participation and learning how to swim contribute to children's development of health and social and psychological welfare (3, 4). On the contrary, swimming is considered the leading activity related to drowning (5). According to the World Health Organization (WHO), aquatic events are the third leading cause of unintentional injury and death worldwide, with an estimated 236,000 annual deaths attributed to drowning (6). It is critical to preserve one's safety in and around water, and it requires a high level of awareness. Children's safety, in particular, in both open and confined waters, is essential for individuals and the community, and factors affecting the safety of children need to be identified for preventive measures to be taken.

Numerous studies indicate that drowning indeed represents a threat to children (5, 7–11). In highly developed countries, both fatal and non-fatal aquatic events (NFAE) are more frequent in public pools (12). However, there is insufficient qualitative data regarding the swimming competence (SC) of young children and adolescents. Analysis of current literature shows that existing methods for assessment of children's SC are rather limited. Often, the examination does not assess a range of skills such as swimming distance or basic aquatic survival skills. For example, previously, SC was evaluated based on their reported maximum swimming distance (13, 14) or subjective swimming skills evaluation (9, 15). Previous studies revealed that SC could be influenced by gender (13), Swimming Experience (2), Age (16), Socio-Economic Status (17), Swimming Location and Accessibility (18).

A recent study by Chan, Lee, and Hamilton (18), shows a positive correlation and prediction of demographic factors (age, sex, school grade, parent education level, and family income) with SC. Also, treading water seemed to be negatively linked with NFAE, meaning that better treading skills are correlated to fewer accidents. However, a certain gap for further investigation of these global problems is needed, especially among the European population. The primary reason for evaluating S.C. is its connection with water safety and fewer NFCA or drowning cases. In addition, a case-control study among the U.S. population found a favorable relationship between swimming lessons and lower drowning risk in young children (8). Also, best to the author's knowledge, none of the previous studies evaluated SC among the Serbian population. Therefore, this study aimed to evaluate SC and examine types of SC in Serbian children.

## Methods

### Participants

All registered local primary schools in Vojvodina, Serbia, were questioned for this study. There were no specific inclusion or exclusion criteria. A total of 2,778 students from first to eighth grade participated in this study. Students incorporated in the study were 10.73 ± 2.1 years old, of which 1,324 (47.7%) were females and 1,454 (52.3%) males. For more demographic characteristics, see Table 1A.

**Table 1A:** Descriptive characteristics of the sample.

	***N*** **(%)**	**Mean (SD)**	**95% CI**
Demographic information		
Age		10.73 (2.1)	10.64–10.81
Sex		
Female	1,324 (47.7)	
Male	1,454 (52.3)	
School grade		
Primary 1	281 (10.1)	
Primary 2	317 (11.4)	
Primary 3	342 (12.3)	
Primary 4	324 (11.7)	
Primary 5	427 (15.4)	
Primary 6	414 (14.9)	
Primary 7	372 (13.4)	
Primary 8	301 (10.8)	
Parent highest education level		
Secondary or lower	987 (26.8)	
Post–school training college	319 (8.7)	
University (undergraduate)	1,082 (29.4)	
University (postgraduate)	390 (10.6)	
Family income		
Low	201 (5.5)	
Middle	1,483 (40.3)	
High	233 (6.3)	
I don't want to declare myself	861 (23.4)	
Swimming experience		
Age to start learning swimming		5.29 (2.41)	5.20–5.38
Years of swimming		5.44 (3.01)	5.32–5.55
Frequency of swimming (times per month)		2.10 (4.65)	1.93–2.28
Duration of swimming (mins per session)		24.71 (34.135)	23.43–25.98
Winter swimming	831 (30.1)		
Swimming location		
Public swimming pools	2,225 (80.6)	
School swimming pools	49 (1.8)	
Club swimming pools	538 (19.5)	
Estate swimming pools	1,001 (36.3)	
Beaches	2,359 (85.4)	
Swimming accessibility		
Public swimming pools	2,526 (91.5)	
School swimming pools	36 (1.3)	
Club swimming pools	687 (24.9)	
Estate swimming pools	1,031 (37.3)	
Beaches	2,083 (75.4)	
Learning experience		
Parent/guardian	1,751 (63.4)	
Swimming club	691 (25.0)	
Government class	22 (0.8)	
Private coach	115 (4.2)	
School/PE teacher	11 (0.5)	
Sibling	67 (2.5)	
Friend	26 (1.0)	
Relative	36 (1.3)	
Grandparent	68 (2.5)	
Non–fatal aquatic event		
Cuts/bruises	230 (6.34)	
Out of breath	56 (1.48)	
Fatigue	205 (5.46)	
Non/fatal drowning	26 (0.88)	
Muscle cramp	75 (2.32)	
Anxiety/panic	59 (1.59)	

### Study design

The study was conducted using a cross-sectional design utilizing a parent-assisted self-reported questionnaire (19). In our study, questionnaire was translated to Serbian language in order for all participants/parents to understand it easily. The questionnaires were not validated in the Serbian language; it is validated only in their original form (19). The participants took around 30 min to complete the questionnaire, which consisted of swimming and demographic-related items. The study was conducted in accordance with Helsinki Declaration and obtained approval from the Ethical Committee of the University of Novi Sad, Serbia (Ref. No. 46-06-02/2020-1). All participants were instructed to complete the questionnaire honestly, and from all parents, written informed authorization to participate was obtained.

### Demographic factors

Several demographic information was collected: age of children, sex, and grade. Parents reported their level of education (recorded as the highest level) and economic status (Table 1B).

**Table 1B:** Descriptive characteristics of the sample.

**Swimming competence index**						
**Maximum swimming distance**		**General**	**Front crawl**	**Breaststroke**	**Backstroke**	**Butterfly**
Very weak (0–4.99 m) (%)		4.30	7.10	6.40	7.10	7.70
Weak (5–12.49 m) (%)		10.55	15.10	13.00	16.30	13.55
Beginner (12.5–24.99 m) (%)		12.15	4.55	11.45	8.75	5.00
Intermediate (25–49.99 m) (%)		22.70	15.70	21.25	14.80	7.45
Good (50–99.99 m) (%)		18.65	12.70	17.40	11.10	4.30
Excellent (<100 m) (%)		19.10	14.00	16.90	13.30	5.25
Mean distance (m)		100.87	77.25	79.30	58.92	21.18
Swimming skills	Poolside kicking	Kicking with kickboard	Holding breath underwater	Floating	Treading water	Swimming underwater
Able	97.20%	91.65%	93.45%	87.00%	90.30%	83.00%
Not able	2.80%	8.35%	6.55%	13.00%	9.70%	17.00%

### Swimming competence

SC was assessed through an SC questionnaire designed by Chan et al. (19). This questionnaire is specifically designed for children to be completed with or without the help of their parents/guardians. Maximum swimming distance (in meters) was reported as distance without resting and using a general stroke. Subjects were able to do any swimming technique that they preferred.

### Level of swimming experience

Swimming experience level was obtained through several questions related to swimming frequency (calculated as how many times they swim in 1 month), duration (calculated in minutes per session), winter swimming participation (1 = yes and 0 = no), age of learning swimming and swimming experience (calculated in years).

### Swimming location and accessibility

For swimming location and accessibility, participants declared whether they had access to swimming locations in a checklist of typical swimming sites. Responses were registered and scored as 1 = yes and 0 = no, relying on the location and access to the pool.

### Learning experience

Participants conveyed their learning experience by selecting from a list of key factors/persons, where they responded if they received the instructions from them. Answers were coded 1 = received swimming instruction and 0 = did not receive swimming instruction.

### Non-fatal aquatic events

To examine NFAE, participants answered by circling the questions associated with accidents or injury during some aquatic events. Answers were coded 1 = existence of NFAE and 0 = absence of NFAE.

### Data analysis

Firstly, descriptive statistics were calculated as means and standard deviation for the number of NFAE and SC. After checking the normality of the distribution, the data for SC data was not distributed normally; the 95% CIs of the descriptive statistics of the variables were estimated by bias-corrected and accelerated bootstrapping with a total of 1.000 resamplings (20). Then, logistic regression and hierarchical multiple regression were used to evaluate if the factors and SC and NFAE were associated. The analyses were carried out by using SPSS® software version 24.0 (SPSS, Inc., Chicago, Illinois, USA). Regarding the hierarchical linear multiple regression model, Demographic Factors were presented as independent variables in Step 1. Whereas, Swimming Location, Swimming Accessibility, and Learning Experience were introduced in Step 2 in separate models. For the hierarchical logistic regression model, demographic factors were placed in Step 1; Swimming Experience was introduced in Step 2; while SC, Swimming Location, Swimming Accessibility, and Learning Experience were placed in Step 3.

## Results

Results from our study showed that participants could swim in general 116.41 meters for males or 84.25 meters for females (Table 2). Also, it was established that distance covered increased with the age of the participants. More than 80% of the participants were able to do every given task (e.g., kicking with a kickboard, floating, treading water, etc.) (for details, see Tables 2, 3). Similar trends with swimming distance were noticed in basic skills, where older kids were more successful and able to do given tasks. Furthermore, only a minority of participants reported that they could not swim further than 5 meters using general stroke (37.15%). Similar trends were obeyed in specific swimming techniques.

**Table 2:** Maximum swimming distance by sex and school grade.

		**General**	**Front-crawl**	**Breaststroke**	**Backstroke**	**Butterfly**
Sex	Male (*N =* 1,444; M-age = 10.71)	116.41 (99.19–136.89)	95.91 (80.43–113.11)	89.15 (75.07–105.79)	68.32 (56.76–81.05)	28.00 (22.73–33.45)
	Female (*N =* 1,308; M-age = 10.74)	84.25 (69.31–101.64)	55.20 (41.30–71.54)	67.01 (54.62–82.68)	47.07 (37.19–60.97)	12.83 (10.03–15.73)
School grade
	Primary 1 (*N =* 277; M-age = 7.11)	18.06 (13.38–23.61)	17.66 (9.69–27.66)	15.78 (12.36–19.67)	9.31 (6.20–12.75)	3.53 (1.50–5.84)
	Primary 2 (*N =* 315; M-age = 8.07)	31.99 (22.11–46.78)	22.21 (12.59–36.72)	31.66 (20.48–50.33)	20.03 (10.50–35.33)	5.13(2.76–8.37)
	Primary 3 (*N =* 341; M-age = 9.12)	50.41 (36.34–68.26)	40.20 (27.75–54.07)	43.98 (28.92–63.51)	26.31 (18.48–35.73)	10.06 (6.53–14.11)
	Primary 4 (*N =* 319; M-age = 10.10)	66.49 (50.94–84.22)	43.55 (31.30–56.92)	53.29 (41.18–67.40)	35.30 (25.99–46.26)	13.17 (9.44–17.96)
	Primary 5 (*N =* 422; M-age = 11.13)	122.01 (94.44–154.44)	88.55 (66.08–114.58)	100.76 (73.28–131.99)	75.09 (57.03–94.77)	27.08 (18.02–37.70)
	Primary 6 (*N =* 412; M-age = 12.20)	143.75 (114.41–178.30)	107.56 (81.67–139.29)	111.15 (89.29–135.55)	79.23 (60.97–100.52)	29.02 (21.20–38.45)
	Primary 7 (*N =* 366; M-age = 18.08)	170.65 (115.01–244.59)	133.01 (83.93–196.83)	108.17 (79.64–153.47)	99.19 (64.87–150.13)	26.1 (17.95–35.84)
	Primary 8 (*N =* 300; M-age = 14.06)	172.14 (128.64–221.34)	136.14 (96.07–185.73)	140.45 (103.22–185.66)	101.54 (74.79–132.09)	46.83 (30.41–67.19)

*Values are mean (95% bias-corrected and accelerated bootstrap confidence interval). Maximum swimming distance indicates the maximum swimming distance participants could swim without any assistance or rest. “General” indicates the maximum swimming by any stroke or combination of strokes. M-age, mean age of the category*.

**Table 3:** Swimming skills by sex and school grade.

**Swimming skills**		**Poolside** **kicking**	**Kicking with** **kickboard**	**Holding breath** **underwater**	**Floating**	**Treading** **water**	**Swimming** **underwater**
Sex
Male	Able	1,414 (97.58)	1,337 (92.27)	1,371 (94.62)	1,258 (86.82)	1,318 (90.96)	1,228 (84.75)
	Not able	35 (2.42)	112 (7.73)	78 (5.38)	191 (13.18)	131 (9.04)	221 (15.25)
Female	Able	1,270 (96.80)	1,194 (91.01)	1,208 (92.07)	1,144 (87.20)	1,175 (89.56)	1,063 (81.02)
	Not able	42 (3.20)	118 (8.99)	104 (7.93)	168 (12.80)	137 (10.44)	249 (18.98)
School grade
Primary 1	Able	256 (91.76)	215 (77.06)	220 (78.85)	182 (65.23)	204 (73.12)	162 (58.06)
	Not able	23 (8.24)	64 (22.94)	59 (21.15)	97 (34.77)	75 (26.88)	117 (41.94)
Primary 2	Able	301 (95.56)	275 (87.30)	278 (88.25)	253 (80.32)	264 (83.31)	217 (68.89)
	Not able	14 (4.44)	40 (12.70)	37 (11.75)	62 (19.68)	51 (16.19)	98 (31.11)
Primary 3	Able	337 (98.54)	319 (93.27)	321 (93.86)	290 (84.80)	301 (88.01)	278 (81.29)
	Not able	5 (5.07)	23 (24.66)	21 (22.37)	52 (61.32)	41 (46.58)	64 (78.73)
Primary 4	Able	315 (98.44)	292 (91.25)	308 (96.25)	282 (88.13)	295 (92.19)	264 (82.50)
	Not able	5 (5.08)	28 (8.75)	12 (3.75)	38 (11.88)	25 (7.81)	56 (17.50)
Primary 5	Able	415 (98.11)	400 (94.56)	407 (96.22)	386 (91.25)	398 (94.09)	384 (90.78)
	Not able	8 (1.89)	23 (5.44)	16 (3.78)	37 (8.75)	25 (5.91)	39 (9.22)
Primary 6	Able	402 (97.34)	395 (95.64)	396 (95.88)	388 (93.95)	399 (96.61)	376 (91.04)
	Not able	11 (2.66)	18 (4.36)	17 (4.12)	25 (6.05)	14 (3.39)	37 (8.96)
Primary 7	Able	362 (98.10)	350 (94.85)	359 (97.29)	339 (91.87)	345 (93.50)	340 (92.14)
	Not able	7 (1.90)	19 (5.15)	10 (2.71)	30 (8.13)	24 (6.50)	29 (7.86)
Primary 8	Able	296 (98.67)	285 (95.00)	290 (96.67)	282 (94.00)	287 (95.67)	270 (90.00)
	Not able	4 (1.33)	15 (5.00)	10 (3.33)	18 (6.00)	13 (4.33)	30 (10.00)

*Values are N (%)*.

### Prediction of swimming competence

In Step 1. demographic factors of gender (β = 0.05, *p* < 0.01), education level (β = 0.06, *p* < 0.01) and age (β = 0.171, *p* < 0.01) were significant SC predictors (R2 = 0.04). In Step 2, following variables were associated with SC: swimming location (ΔR2 = 0.06, *p* < 0.01), swimming experience (ΔR2 = 0.16, *p* < 0.01), swimming accessibility (ΔR2 = 0.05, *p* < 0.01), and learning experience (ΔR2 = 0.03, *p* < 0.01) (total R2 = 0.26 to 0.47, *p* < 0.01). Result are displayed in Table 4.

**Table 4:** Results of hierarchical linear multiple regression models.

**Dependent variable** = **swimming competence**								
**Step**	**Independent variables**	**β**	**95% CI of B**	***F***	**ΔF**	***R* ^2^**	**Δ*R*^2^**	**VIF**
Demographic factors								
1	Age	0.171**	20.690 to 32.551	25.378**	N/A	0.036	N/A	1.027
	Sex	−0.049**	−60.951 to −8.378		1.000
	Parents' highest education	0.059**	8.130 to 27.842		1.048
	Family Income	0.037	0.591 to 25.340		1.038
Swimming experience
2	Years of swimming	−0.054**	−11.433 to −4.441	66.038**	102.928**	0.162	0.126	1.028
	Frequency of swimming	0.304**	14.834 to 32.525					1.482
	Swimming duration	0.051*	−0.014 to 1.067					1.443
	Winter swimming	−0.044*	−60.303 to −4.562					1.294
Swimming location								
2	Public swimming pool	−0.042*	−58.707 to −13.848	19.535**	14.478**	0.060	0.025	1.049
	School swimming pool	0.025	2.361 to 125.652					1.036
	Club swimming pool	−0.139**	−165.500 to −75.154					1.052
	Estate swimming pool	0.031	−1.160 to 47.519					1.039
	Beach	0.046*	1.239 to 97.989					1.053
Swimming accessibility								
2	Public swimming pool	0.003	−29.380 to 44.213	16.989**	10.057**	0.053	0.017	1.056
	School swimming pool	−0.004	−106.842 to 68.074		1.023
	Club swimming pool	−0.117**	−128.647 to −60.982		1.036
	Estate swimming pool	0.044*	6.093 to 55.270		1.039
	Beach	0.047*	3.550 to 76.367		1.062
Learning experience								
2	Learning experience	−0.004	−5.624 to 3.648	20.194**	0.048**	0.035	0.000	1.003

Step 1 was identical throughout the three models. CI of B, 95% bias-corrected and accelerated bootstrap confidence. VIF, variance inflation factor. Swimming duration, duration of each swimming session in minutes. Learning experience, “Learn swimming from whom?”. PE teacher, physical education teacher. *p <0.05; **p <0.01; N/A - not applicable.

### Prediction of non-fatal aquatic events

NFAE estimation revealed that the demographic factor of age (OR = 1.15, *p* < 0.01) was the only significant predictor in Step 1. Further, in Step 2 and Step 3. there were no statistically significant factors. The results are displayed in Table 5.

**Table 5:** Results of hierarchical logistic regression models.

**Dependent variable** = **non-fatal events**
**Step**	**Variables**	**Odds ratio**	**95% CI of B**	**WALD**	***X* ^2^**	***R* ^2^**	**Δ*R*^2^**
Demographic factors							
1	Age	1.151**	1.109–1.195	54.651	58.469**	0.029	N/A
	Sex	1.102	0.939–1.294	1.422	
	Parents' highest education	1.027	0.956–1.103	0.535	
	Family income	1.005	0.927–1.091	0.018	
Swimming experience							
2	Years of swimming	0.997	0.963–1.031	0.040	58.568**	0.029	–
	Frequency of swimming	1.001	0.980–1.022	0.011	
	Swimming duration	1.000	0.997–1.003	0.018	
	Winter swimming	1.000	0.821–1,219	0.000	
Swimming competence							
3	General	1.000	0.999–1.000	0.213	11.433	0.034	0.005
	Breaststroke	1.000	0.999–1.000	0.427	
	Front-crawl	1.000	0.999–1.001	0.330	
	Backstroke	1.000	0.999–1.001	0.143	
	Butterfly	1.000	0.998–1.001	0.352	
	Swimming underwater	1.215	0.922–1.602	1.908	
	Floating	1.114	0.816–1.522	0.465	
	Poolside kicking	1.541	0.872–2.724	2.212	
	Kickboard kicking	1.133	0.773–1.661	0.410	
	Holding breath underwater	0.816	0.538–1.238	0.917	
	Treading water	0.905	0.628–1.303	0.289	
Swimming location							
3	Public swimming pool	1.082	0.874–1.339	0.527	64.141**	0.032	0.003
	School swimming pool	1.257	0.651–2.430	0.465	
	Club swimming pool	1.186	0.940–1.496	2.074	
	Estate swimming pool	0.943	0.796–1.117	0.465	
	Beach	1.108	0.876–1.401	0.735	
Swimming accessibility							
3	Public swimming pool	1.029	0.764–1.384	0.035	62.323**	0.031	0.002
	School swimming pool	1.264	0.597–2.678	0.375	
	Club swimming pool	1.137	0.929–1.390	1.551	
	Estate swimming pool	0.925	0.782–1.095	0.815	
	Beach	1.050	0.866–1.272	0.244	
Learning experience							
3	Learning experience	0.996	0.945–1.049	0.027	59.267**	0.030	0.001

Controls are age, gender, grade, parents' education, income and years, frequency, duration of swimming, and winter swimming. Steps 1 and 2 were identical throughout the three models. R2, Nagelkerke R-squared. 95% CI of EXP (B), 95% confidence interval of the odds ratio. Learning experience, “Learn swimming from whom?”. PE teacher, physical education teacher. **p <0.01; N/A - not applicable.

## Discussion

The present study aimed to explore the SC and NFAE and their interconnected factors in a sample of primary school pupils in Vojvodina, Serbia. Study results explore the current understanding of children's SC. The connections between SC and NFAE are significant given that the existing literature has predominantly concentrated on factors linked to fatal drowning (7, 21–23). Therefore, these results could help expand further knowledge of this specific topic and help contribute to future projects to address SC in the primary school population.

Results in the current study differ from the previously available data. More than 80% of the study sample were capable of doing every given task. In addition, only 35% of them reported that they could not swim the 5 m distance. On the opposite, in Fife and Goldoft (24) study, nearly 38 % of youths under 14 were declared non-swimmers, and 37% of adults had registered as very limited SC (13). In addition to these results, 37% of the Hong Kong primary school pupils were underlined as weak swimmers with a low level of SC (18). Compared to previous studies, cultural diversity should also be considered as one of the factors for the possible difference in the results. However, even the results from the present study show much higher levels of SC; the initiatives must be taken in a mission to promote swimming skills.

Present results displayed that pupils with greater SC were predominantly from higher income and/or higher education levels families. Previous research unequivocally indicates that children from families with lower economic or educational status generally show lower participation in sports and typically have lower coach-led activity levels (17, 25), which corresponds with results obtained from our sample. Similar findings were found in Chan, Lee, and Hamilton (18) study. These results indicate that special attention should be paid to enabling children's education from these families. Present results also revealed that boys had significantly better SC compared to girls. Although small, gender differences are compatible with prior investigations (13, 26). Other variables in step two also shown significant correlation with SC. Swimming location and accessibility of swimming facilities (public, school, and club swimming pools) were correlated positively with swimming competence. Similar findings were observed in the study by Chan et al. (18), where they highlighted the importance of swimming facilities and their availability to children SC.

Although some of the factors such as years of swimming, swimming frequency and learning experience were significant predictors of children's SC (2, 24), but these factors were not significant predictors of NFAE. In previous studies, only the duration of each swimming session was found to correlate positively with NFAE (2, 24). However, in the present study, age was the only significant factor for prediction of NFAE. Similar to these findings, other studies have similar conclusions that younger children had more chances for NFAE (9, 18). Interestingly there was no difference in risk of NFAE based on pupils' gender. For example, in the study by Chan, Lee, and Hamilton (18), males had greater SC, but there was no difference in NFAE in genders. Consequently, the gender differences should be further explored to develop an awareness of dangerous aquatic behaviors.

Another interesting finding was that none of the demographic characteristics or variables in swimming experience and SC, swimming location, swimming accessibility, or learning experience were influential predictors of NFAE. These results can partially be explained because most children usually participated/visited swimming areas such as pools supervised by lifeguards, which could influence the low rate of NFAE.

Although results from this study are promising, drowning still represents a major concern. Firstly, children from families with lower income should be included in educational and practical sessions, with special attention addressed to water safety. Secondly, national and community programs should be developed in such a way as to educate parents and coaches about the importance of water safety. We highly recommend parents/guardians teach children to swim as early as possible. The development of the infrastructure in schools, with access to the swimming pool and an increased number of public swimming pools, can significantly influence the rate of drowning and NFAE and increase SC.

We must highlight that this study had several limitations. First, the usage of questionnaires, retrospective measures, and self-report data of SC and NFAE may increase worries about social utility and response bias (19, 27). On the other hand, this questionnaire showed high correlations with the coaches' attitudes about SC children (18). Secondly, this study was conducted on a narrow population of one province in Serbia. Most of the children/parents surveyed are primarily from cities, which leaves the question of the actual situation of SC children from non-urban areas. In the end, future studies should consider selecting longitudinal studies with a mission to better understand and confirm available findings. Regardless of limitations, this study was the first to investigate swimming competence in Vojvodina and Serbia. Further, the research was conducted on considerably large sample size, and statistical methods were divided into steps to understand the results of this important topic better.

## Conclusion

The results of our study indicate fundamental data on SC and NFAE. Children from wealthier families generally have more experience, knowledge, and skills related to water safety. Further, older children have higher competency, knowledge, and a lower volume of NFAE. Therefore, the education of children and parents must be a priority in the field of safety in and around water facilities or open water places. In addition, infrastructure and general accessibility of facilities such as swimming pools, landscaped beaches, and aqua parks must be prioritized by the community and governments. Furthermore, educating children in non-urban environments about water safety is one of the key long-term goals, although there are lack information on their swimming competencies. In the future, national education trainers programs must be prioritized with a primary mission to transfer knowledge and skills in swimming and water safety.

## Data availability statement

The original contributions presented in the study are included in the article/supplementary material, further inquiries can be directed to the corresponding author.

## Ethics statement

The studies involving human participants were reviewed and approved by Ethical Committee of the University of Novi Sad, Serbia (Ref. No. 46-06-02/2020-1). Written informed consent to participate in this study was provided by the participants' legal guardian/next of kin.

## Author contributions

AS-G, BA, NT, and JF-L were involved in study conception and design and wrote the first draft of the manuscript. MR, VŠ, NZ, AS-G, and JF-L collected the data and analyzed the data. AB, PD, and JC-G revised, edited, and approved the final manuscript. All authors contributed to the article and approved the submitted version.

## Funding

This work has been supported by the Serbian Ministry of Education, Science, and Technological Development and the Provincial Secretariat for Higher Education and Scientific Research (142-451-2594).

## Conflict of interest

The authors declare that the research was conducted in the absence of any commercial or financial relationships that could be construed as a potential conflict of interest.

## Publisher's note

All claims expressed in this article are solely those of the authors and do not necessarily represent those of their affiliated organizations, or those of the publisher, the editors and the reviewers. Any product that may be evaluated in this article, or claim that may be made by its manufacturer, is not guaranteed or endorsed by the publisher.

## Abbreviations

NFAE: non-fatal aquatic events
SC: swimming competence.
